# 7-Meth­oxy­penta­cyclo­[5.4.0.0^2,6^.0^3,10^.0^5,9^]undec­ane-8,11-dione

**DOI:** 10.1107/S2414314620013802

**Published:** 2020-10-20

**Authors:** Sambasivarao Kotha, Saima Ansari, Subba Rao Cheekatla

**Affiliations:** a Department of Chemistry, Indian Institute of Technology Bombay, Powai, Mumbai - 400076, India; Goethe-Universität Frankfurt, Germany

**Keywords:** crystal structure, Diels–Alder reaction, intra­molecular [2 + 2] photo­cyclo­addition, penta­cyclo [5.4.0.0^2,6^.0^3,10^.0^5,9^]undeca­ne

## Abstract

The structure of 7-meth­oxy­penta­cyclo­[5.4.0.0^2,6^.0^3,10^.0^5,9^]undecane-8,11-dione, C_12_H_12_O_3_, at 150 K has monoclinic (*P*2_1_/*c*) symmetry. The penta­cyclo­undecane cage compound is composed of four five-membered rings, a planar four-membered ring and a six-membered ring in a boat conformation fused into a closed strained-cage framework. All of the five-membered rings adopt an envelope conformation.

## Abstract 2

The crystal structure of a meth­oxy-substituted Cookson’s dione derivative is presented.

## Graphical abstract

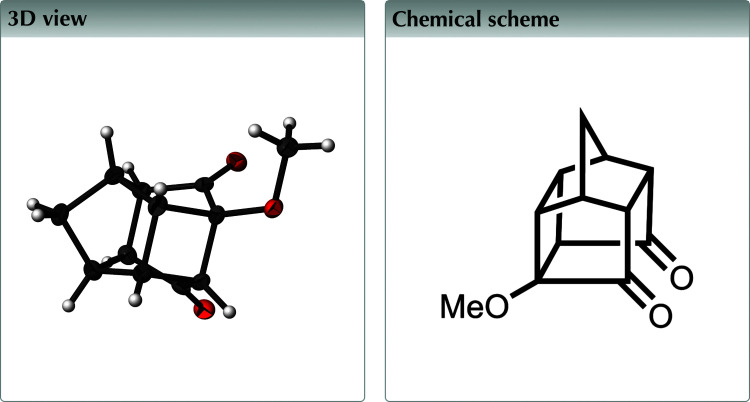

## Structure description

Polycyclic cage hydro­carbons act as valuable synthons in pharmaceutical and medicinal chemistry (Bisetty *et al.*, 2006 ▸; Geldenhuys, *et al.*, 2005 ▸). They are also useful candidates in energetic materials (Zhang *et al.*, 2018 ▸). Most of the cage systems display functions in supra­molecular chemistry and asymmetric catalysis (Gharpure *et al.*, 2013 ▸). Oxygenated cage compounds show significant biological activity (Oliver *et al.*, 1991 ▸; van Dijk *et al.*, 2008 ▸). In addition, various rearrangement approaches in penta­cyclo­undecane-containing cage frameworks provide an alternative route for the synthesis of biologically relevant frameworks such as *D*_3_-tris­homocubane derivatives that are not available by conventional multi-step synthetic routes (Liu *et al.*, 2001 ▸; Sklyarova *et al.*, 2013 ▸).

**Figure 1:**
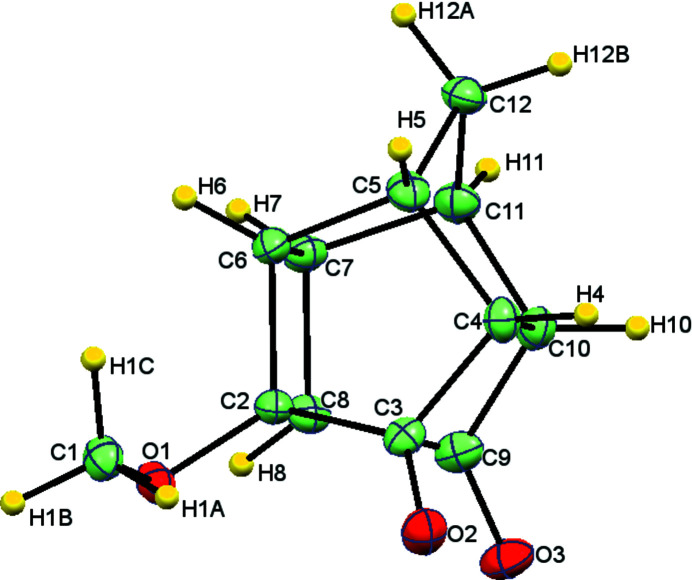
Perspective view of the title compound. Displacement parameters are drawn at the 50% probability level.

The bond angle C1—O1—C2 is 113.86°(16). Notably, the distance between the meth­oxy-substituted carbon atom, C2, and C3 is 1.521 (3) Å while the C8—C9 distance is 1.516 (3) Å. The meth­oxy substitution (presence of electron-donating group) has led to an elongation of the C2—C3 bond. Additionally, all bonds of the cyclo­butane ring are not equal, the observed range being 1.552 (3)–1.579 (3) Å. Thus, a slight distortion is observed after substitution with a meth­oxy group (Fig. 1 ▸) compared to Cookson’s dione skeleton (Linden *et al.*, 2005 ▸).

## Synthesis and crystallization

**Figure 2:**
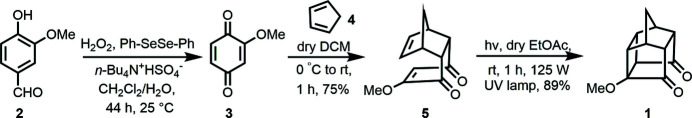
Reaction scheme for the synthesis of the title compound.

The title compound **1** was prepared (Fig. 2 ▸) from quinone **3**, which was derived from the commercially available starting materials 4-hy­droxy-3-meth­oxy­benzaldehyde **2** (vanillin) and cyclo­penta­diene **4** using the reported method (Pratt *et al.*, 1987 ▸) *via* a Diels–Alder reaction and [2 + 2] photo­cyclo­addition as key steps (Mehta *et al.*, 1984 ▸). The Diels–Alder adduct **5** (100 mg, 0.40 mmol, synthesized from quinone **3** *via* Diels–Alder reaction with freshly cracked cyclo­penta­diene **4**) was dissolved in anhydrous ethyl acetate (300 ml) and irradiated in a Pyrex immersion well using a 125 W medium-pressure UV mercury-vapour lamp for 30 min under nitro­gen at room temperature. After conclusion of the reaction as monitored by TLC, the solvent was evaporated under reduced pressure and the crude reaction mixture was purified by silica gel column chromatography using 40% ethyl acetate/petroleum ether as an eluent, which furnished **1** as a colourless solid. The resulting isolated compound was crystallized from petroleum ether and CHCl_3_ (4:1) in a refrigerator by slow evaporation (83 mg, 83%). Colourless crystalline solid, m.p. 89–91°C; (lit. reported m.p. 85°C); ^1^H NMR (500 MHz, CDCl_3_): *δ* = 3.40 (*s*, 3H), 3.24–3.20 (*m*, 1H), 3.08–3.05 (*m*, 1H), 2.93 (*s*, 1H), 2.89 (*s*, 1H), 2.83 (*d*, *J* = 6.4 Hz, 1H), 2.68–2.58 (*m*, 2H), 2.00 (*d*, *J* = 11.4 Hz, 1H), 1.9 (*d*, *J* = 11.4 Hz, 1H) p.p.m. ^13^C NMR (125 MHz, CDCl_3_): *δ* = 210.8, 209.9, 82.1, 54.7, 53.5, 50.8, 48.5, 43.9, 43.2, 41.9, 36.4 p.p.m. HRMS (ESI): *m/z* calculated for C_12_H_12_NaO_3_ [*M* + K]^+^: 243.418; found: 243.415.

## Refinement

Crystal data, data collection and structure refinement details are summarized in Table 1 ▸.

## Supplementary Material

Crystal structure: contains datablock(s) I. DOI: 10.1107/S2414314620013802/bt4099sup1.cif

Structure factors: contains datablock(s) I. DOI: 10.1107/S2414314620013802/bt4099Isup3.hkl

Supporting information file. DOI: 10.1107/S2414314620013802/bt4099Isup3.cml

CCDC reference: 1902538

Additional supporting information:  crystallographic information; 3D view; checkCIF report

## Figures and Tables

**Table 1:** Experimental details

Crystal data	
Chemical formula	C_12_H_12_O_3_
*M*_r_	204.22
Crystal system, space group	Monoclinic, *P*2_1_/*c*
Temperature (K)	150
*a*, *b*, *c* (Å)	6.3136 (2), 11.6138 (5), 12.6330 (5)
β (°)	95.292 (3)
*V* (Å^3^)	922.37 (6)
*Z*	4
Radiation type	Mo *K*α
μ (mm^−1^)	0.11
Crystal size (mm)	0.34 × 0.28 × 0.23

Data collection	
Diffractometer	Oxford Diffraction Xcalibur-S
Absorption correction	Multi-scan (*CrysAlis RED*; Oxford Diffraction, 2008 ▸)
*T*_min_, *T*_max_	0.965, 0.976
No. of measured, independent and observed [*I* > 2σ(*I*)] reflections	4686, 1620, 1432
*R*_int_	0.020
(sin θ/λ)_max_ (Å^−1^)	0.594

Refinement	
*R*[*F* ^2^ > 2σ(*F* ^2^)], *wR*(*F* ^2^), *S*	0.048, 0.111, 1.10
No. of reflections	1620
No. of parameters	137
H-atom treatment	H-atom parameters constrained
Δρ_max_, Δρ_min_ (e Å^−3^)	0.31, −0.19

Computer programs: *CrysAlis CCD* and *CrysAlis RED* (Oxford Diffraction, 2008 ▸), *SHELXT* (Sheldrick, 2015*a* ▸), *SHELXL* (Sheldrick, 2015*b* ▸) and *OLEX2* (Dolomanov *et al.*, 2009 ▸).

## full crystallographic data

### Crystal data

**Table:**

C_12_H_12_O_3_	*F*(000) = 432
*M**_r_* = 204.22	*D*_x_ = 1.471 Mg m^−^^3^
Monoclinic, *P*2_1_/*c*	Mo *K*α radiation, λ = 0.71073 Å
*a* = 6.3136 (2) Å	Cell parameters from 1649 reflections
*b* = 11.6138 (5) Å	θ = 3.3–32.8°
*c* = 12.6330 (5) Å	µ = 0.11 mm^−^^1^
β = 95.292 (3)°	*T* = 150 K
*V* = 922.37 (6) Å^3^	Block, colourless
*Z* = 4	0.34 × 0.28 × 0.23 mm

### Data collection

**Table:**

Oxford Diffraction Xcalibur-S diffractometer	1432 reflections with *I* > 2σ(*I*)
Detector resolution: 15.9948 pixels mm^-1^	*R*_int_ = 0.020
ω/q–scan	θ_max_ = 25.0°, θ_min_ = 3.7°
Absorption correction: multi-scan (CrysAlis RED; Oxford Diffraction, 2008)	*h* = −7→4
*T*_min_ = 0.965, *T*_max_ = 0.976	*k* = −13→13
4686 measured reflections	*l* = −13→15
1620 independent reflections	

### Refinement

**Table:**

Refinement on *F*^2^	Primary atom site location: dual
Least-squares matrix: full	Hydrogen site location: inferred from neighbouring sites
*R*[*F*^2^ > 2σ(*F*^2^)] = 0.048	H-atom parameters constrained
*wR*(*F*^2^) = 0.111	*w* = 1/[σ^2^(*F*_o_^2^) + (0.0339*P*)^2^ + 1.1811*P*] where *P* = (*F*_o_^2^ + 2*F*_c_^2^)/3
*S* = 1.10	(Δ/σ)_max_ < 0.001
1620 reflections	Δρ_max_ = 0.31 e Å^−^^3^
137 parameters	Δρ_min_ = −0.19 e Å^−^^3^
0 restraints	

### Special details

**Table:**

Geometry. All esds (except the esd in the dihedral angle between two l.s. planes) are estimated using the full covariance matrix. The cell esds are taken into account individually in the estimation of esds in distances, angles and torsion angles; correlations between esds in cell parameters are only used when they are defined by crystal symmetry. An approximate (isotropic) treatment of cell esds is used for estimating esds involving l.s. planes.
Refinement. The H atoms were found in a difference map but refined using a riding model with C—H ranging from 0.98 Å to 1.00 Å and U(H) set to 1.2 *U*_eq_(C) or 1.5 *U*_eq_(C_methyl_).

### Fractional atomic coordinates and isotropic or equivalent isotropic displacement parameters (Å^2^)

**Table:**

	*x*	*y*	*z*	*U*_iso_*/*U*_eq_	
O1	0.5101 (2)	0.05611 (13)	0.88757 (12)	0.0213 (4)	
O2	0.7295 (2)	0.00528 (14)	0.69852 (12)	0.0252 (4)	
O3	0.1795 (2)	−0.10734 (14)	0.62216 (13)	0.0281 (4)	
C1	0.7014 (4)	0.1112 (2)	0.93007 (18)	0.0231 (5)	
H1A	0.8199	0.0856	0.8910	0.035*	
H1B	0.7304	0.0910	1.0053	0.035*	
H1C	0.6852	0.1949	0.9231	0.035*	
C2	0.4378 (3)	0.09282 (18)	0.78544 (17)	0.0183 (5)	
C3	0.5748 (3)	0.06703 (18)	0.69536 (17)	0.0186 (5)	
C4	0.4765 (3)	0.13774 (19)	0.60217 (17)	0.0205 (5)	
H4	0.5664	0.1421	0.5411	0.025*	
C5	0.4380 (3)	0.25570 (19)	0.65426 (17)	0.0219 (5)	
H5	0.5615	0.3100	0.6586	0.026*	
C6	0.3617 (3)	0.21926 (19)	0.76243 (17)	0.0204 (5)	
H6	0.3814	0.2757	0.8223	0.024*	
C7	0.1322 (3)	0.17496 (19)	0.73067 (17)	0.0201 (5)	
H7	0.0168	0.2055	0.7720	0.024*	
C8	0.2040 (3)	0.04885 (19)	0.75312 (17)	0.0202 (5)	
H8	0.1322	0.0060	0.8084	0.024*	
C9	0.2003 (3)	−0.00636 (19)	0.64421 (18)	0.0211 (5)	
C10	0.2401 (3)	0.0924 (2)	0.57020 (18)	0.0225 (5)	
H10	0.2100	0.0735	0.4931	0.027*	
C11	0.1045 (4)	0.1921 (2)	0.60828 (18)	0.0232 (5)	
H11	−0.0461	0.1944	0.5759	0.028*	
C12	0.2344 (4)	0.30022 (19)	0.59218 (18)	0.0239 (5)	
H12A	0.1760	0.3693	0.6250	0.029*	
H12B	0.2528	0.3151	0.5164	0.029*	

### Atomic displacement parameters (Å^2^)

**Table:**

	*U*^11^	*U*^22^	*U*^33^	*U*^12^	*U*^13^	*U*^23^
O1	0.0240 (8)	0.0213 (8)	0.0187 (8)	−0.0036 (7)	0.0019 (6)	0.0028 (6)
O2	0.0209 (8)	0.0287 (9)	0.0265 (9)	0.0030 (7)	0.0040 (6)	−0.0010 (7)
O3	0.0281 (9)	0.0185 (9)	0.0369 (10)	−0.0011 (7)	−0.0014 (7)	−0.0055 (7)
C1	0.0258 (12)	0.0230 (12)	0.0200 (11)	−0.0045 (10)	−0.0006 (9)	−0.0018 (9)
C2	0.0223 (11)	0.0147 (11)	0.0178 (11)	0.0000 (9)	0.0015 (9)	0.0009 (8)
C3	0.0167 (11)	0.0172 (11)	0.0223 (11)	−0.0036 (9)	0.0032 (8)	−0.0021 (9)
C4	0.0188 (10)	0.0250 (12)	0.0182 (11)	−0.0013 (9)	0.0045 (8)	−0.0004 (9)
C5	0.0237 (11)	0.0199 (12)	0.0222 (12)	−0.0044 (9)	0.0026 (9)	0.0015 (9)
C6	0.0260 (11)	0.0162 (11)	0.0194 (11)	−0.0004 (9)	0.0042 (9)	−0.0011 (9)
C7	0.0186 (11)	0.0176 (11)	0.0249 (12)	0.0029 (9)	0.0060 (9)	0.0000 (9)
C8	0.0181 (11)	0.0190 (11)	0.0243 (12)	−0.0009 (9)	0.0065 (8)	0.0031 (9)
C9	0.0130 (10)	0.0202 (12)	0.0292 (12)	0.0001 (9)	−0.0034 (8)	−0.0024 (9)
C10	0.0235 (12)	0.0234 (12)	0.0201 (12)	0.0013 (9)	−0.0009 (9)	−0.0034 (9)
C11	0.0209 (11)	0.0234 (12)	0.0249 (12)	0.0030 (9)	0.0001 (9)	0.0015 (10)
C12	0.0286 (12)	0.0187 (11)	0.0248 (12)	0.0024 (10)	0.0047 (9)	0.0048 (9)

### Geometric parameters (Å, º)

**Table:**

O1—C1	1.427 (3)	C5—C12	1.533 (3)
O1—C2	1.395 (3)	C6—H6	1.0000
O2—C3	1.209 (3)	C6—C7	1.555 (3)
O3—C9	1.210 (3)	C7—H7	1.0000
C1—H1A	0.9800	C7—C8	1.552 (3)
C1—H1B	0.9800	C7—C11	1.553 (3)
C1—H1C	0.9800	C8—H8	1.0000
C2—C3	1.521 (3)	C8—C9	1.516 (3)
C2—C6	1.564 (3)	C9—C10	1.516 (3)
C2—C8	1.579 (3)	C10—H10	1.0000
C3—C4	1.520 (3)	C10—C11	1.543 (3)
C4—H4	1.0000	C11—H11	1.0000
C4—C5	1.549 (3)	C11—C12	1.523 (3)
C4—C10	1.599 (3)	C12—H12A	0.9900
C5—H5	1.0000	C12—H12B	0.9900
C5—C6	1.549 (3)		
			
C2—O1—C1	113.86 (16)	C6—C7—H7	117.0
O1—C1—H1A	109.5	C8—C7—C6	90.88 (16)
O1—C1—H1B	109.5	C8—C7—H7	117.0
O1—C1—H1C	109.5	C8—C7—C11	107.91 (17)
H1A—C1—H1B	109.5	C11—C7—C6	103.36 (17)
H1A—C1—H1C	109.5	C11—C7—H7	117.0
H1B—C1—H1C	109.5	C2—C8—H8	117.0
O1—C2—C3	118.11 (17)	C7—C8—C2	89.57 (16)
O1—C2—C6	121.84 (18)	C7—C8—H8	117.0
O1—C2—C8	111.03 (17)	C9—C8—C2	107.88 (17)
C3—C2—C6	103.41 (17)	C9—C8—C7	104.69 (18)
C3—C2—C8	108.99 (17)	C9—C8—H8	117.0
C6—C2—C8	89.53 (15)	O3—C9—C8	127.7 (2)
O2—C3—C2	127.2 (2)	O3—C9—C10	127.8 (2)
O2—C3—C4	128.1 (2)	C10—C9—C8	104.48 (18)
C4—C3—C2	104.68 (17)	C4—C10—H10	114.1
C3—C4—H4	113.9	C9—C10—C4	107.28 (17)
C3—C4—C5	102.57 (17)	C9—C10—H10	114.1
C3—C4—C10	108.83 (17)	C9—C10—C11	104.33 (18)
C5—C4—H4	113.9	C11—C10—C4	101.91 (17)
C5—C4—C10	102.63 (17)	C11—C10—H10	114.1
C10—C4—H4	113.9	C7—C11—H11	115.2
C4—C5—H5	115.4	C10—C11—C7	101.50 (17)
C4—C5—C6	101.95 (17)	C10—C11—H11	115.2
C6—C5—H5	115.4	C12—C11—C7	103.08 (18)
C12—C5—C4	103.79 (17)	C12—C11—C10	104.82 (18)
C12—C5—H5	115.4	C12—C11—H11	115.2
C12—C5—C6	103.25 (17)	C5—C12—H12A	112.7
C2—C6—H6	117.4	C5—C12—H12B	112.7
C5—C6—C2	107.77 (17)	C11—C12—C5	95.17 (17)
C5—C6—H6	117.4	C11—C12—H12A	112.7
C5—C6—C7	102.77 (17)	C11—C12—H12B	112.7
C7—C6—C2	90.01 (16)	H12A—C12—H12B	110.2
C7—C6—H6	117.4		
			
O1—C2—C3—O2	10.0 (3)	C5—C4—C10—C11	0.4 (2)
O1—C2—C3—C4	−168.95 (17)	C5—C6—C7—C8	108.30 (17)
O1—C2—C6—C5	141.85 (19)	C5—C6—C7—C11	−0.3 (2)
O1—C2—C6—C7	−114.7 (2)	C6—C2—C3—O2	147.9 (2)
O1—C2—C8—C7	124.27 (18)	C6—C2—C3—C4	−31.0 (2)
O1—C2—C8—C9	−130.43 (18)	C6—C2—C8—C7	0.06 (16)
O2—C3—C4—C5	−134.1 (2)	C6—C2—C8—C9	105.37 (18)
O2—C3—C4—C10	117.7 (2)	C6—C5—C12—C11	−53.4 (2)
O3—C9—C10—C4	−111.3 (2)	C6—C7—C8—C2	−0.06 (16)
O3—C9—C10—C11	141.1 (2)	C6—C7—C8—C9	−108.45 (17)
C1—O1—C2—C3	65.0 (2)	C6—C7—C11—C10	74.92 (19)
C1—O1—C2—C6	−64.9 (2)	C6—C7—C11—C12	−33.4 (2)
C1—O1—C2—C8	−168.08 (17)	C7—C8—C9—O3	−155.1 (2)
C2—C3—C4—C5	44.8 (2)	C7—C8—C9—C10	27.8 (2)
C2—C3—C4—C10	−63.4 (2)	C7—C11—C12—C5	52.89 (19)
C2—C6—C7—C8	0.06 (16)	C8—C2—C3—O2	−117.9 (2)
C2—C6—C7—C11	−108.58 (17)	C8—C2—C3—C4	63.2 (2)
C2—C8—C9—O3	110.5 (2)	C8—C2—C6—C5	−103.48 (18)
C2—C8—C9—C10	−66.6 (2)	C8—C2—C6—C7	−0.06 (16)
C3—C2—C6—C5	5.9 (2)	C8—C7—C11—C10	−20.4 (2)
C3—C2—C6—C7	109.36 (17)	C8—C7—C11—C12	−128.80 (18)
C3—C2—C8—C7	−103.97 (18)	C8—C9—C10—C4	65.8 (2)
C3—C2—C8—C9	1.3 (2)	C8—C9—C10—C11	−41.8 (2)
C3—C4—C5—C6	−39.4 (2)	C9—C10—C11—C7	37.7 (2)
C3—C4—C5—C12	−146.43 (17)	C9—C10—C11—C12	144.74 (18)
C3—C4—C10—C9	−0.7 (2)	C10—C4—C5—C6	73.49 (19)
C3—C4—C10—C11	108.63 (19)	C10—C4—C5—C12	−33.6 (2)
C4—C5—C6—C2	20.5 (2)	C10—C11—C12—C5	−53.0 (2)
C4—C5—C6—C7	−73.64 (19)	C11—C7—C8—C2	104.29 (18)
C4—C5—C12—C11	52.71 (19)	C11—C7—C8—C9	−4.1 (2)
C4—C10—C11—C7	−73.82 (19)	C12—C5—C6—C2	128.00 (18)
C4—C10—C11—C12	33.2 (2)	C12—C5—C6—C7	33.8 (2)
C5—C4—C10—C9	−108.86 (19)		
